# Prophylactic transarterial embolization after endoscopic hemostasis in patients with non-variceal upper gastrointestinal bleeding - is it time to act?

**DOI:** 10.3389/fradi.2026.1719448

**Published:** 2026-04-02

**Authors:** Francesco Tiralongo, Roberto Minici, Makoto Taninokuchi Tomassoni, Corrado Ini', Davide Giuseppe Castiglione, Francesco Vacirca, Cristina Mosconi, Stefania Tamburrini, Giuseppe Messina, Emanuele David, Pietro Valerio Foti, Stefano Palmucci, Antonio Basile

**Affiliations:** 1 Radiology Unit 1, Department of Medical Surgical Sciences and Advanced Technologies “GF Ingrassia”, University Hospital Policlinico “G. Rodolico-San Marco”, University of Catania, Catania, Italy; 2 Radiology Unit, Department of Experimental and Clinical Medicine, University Hospital Mater Domini, Magna Graecia University of Catanzaro, Catanzaro, Italy; 3 Department of Radiology, IRCCS Azienda Ospedaliero Universitaria Di Bologna, Bologna, Italy; 4 Department of Radiology, Ospedale del Mare, ASL NA1 Centro, Naples, Italy; 5 UOSD “IPTRA”, Department of Medical Surgical Sciences and Advanced Technologies “GF Ingrassia”, University Hospital Policlinico “G. Rodolico-San Marco”, University of Catania, Catania, Italy

**Keywords:** angiography, digital subtraction; embolization, therapeutic; gastrointestinal hemorrhage; prophylactive transarterial embolization; radiology, interventional; upper gastrointestinal bleeding

## Abstract

**Background:**

Non-variceal upper gastrointestinal bleeding (NVUGIB) continues to present a significant clinical burden due to rebleeding after apparently successful endoscopic hemostasis, particularly in ulcers overlying large-caliber arterial territories. Prophylactic transarterial embolization (pTAE) has been proposed as a strategy to prevent rebleeding in high-risk patients. This mini-review evaluates the evidence for pTAE after successful endoscopic control in NVUGIB, focusing on patient selection, technical approaches, outcomes, and complications.

**Methods:**

A literature search of PubMed and Scopus (January 2010–September 2025) was conducted, yielding 10 studies (two randomized trials, three prospective, and five retrospective) evaluating pTAE. Only studies addressing prophylactic, not empiric, embolization were included.

**Results:**

Evidence suggests that pTAE is technically feasible and generally safe when guided by ulcer location, size (≥15–20 mm), Rockall score (≥5), and arterial territory (GDA or LGA). While randomized trials did not show overall superiority in intention-to-treat analyses, per-protocol data and observational studies suggest reduced rebleeding and a lower need for surgical rescue in well-selected patients. Complications are infrequent when standardized techniques and early timing (≤24 h) are applied.

**Conclusion:**

Routine pTAE is not supported by current guidelines or RCT-level evidence. However, in anatomically and clinically high-risk ulcers, pTAE may offer meaningful benefits. Further multicenter randomized trials with uniform protocols are warranted to clarify its role and optimize patient selection.

## 1 Introduction

Non-variceal upper gastrointestinal bleeding (NVUGIB) remains a frequent reason for emergency admission and early mortality despite timely resuscitation, endoscopy, and usage of high-dose proton-pump inhibitors; recurrent hemorrhage after apparently successful endoscopic hemostasis is the main driver of adverse outcomes and resource use (1).

Rebleeding occurs predominantly in ulcers located over large-caliber arterial territories, particularly the posterior duodenal bulb adjacent to the gastroduodenal artery (GDA) and the lesser curvature or angulus along the course of the left gastric artery (LGA), where substantial arterial inflow and well-developed collateral networks may limit the long-term effectiveness of endoscopic therapy (1).

Endovascular management is established as a rescue option once endoscopic control fails or bleeding recurs. In comparative analysis vs. surgery, transarterial embolization (TAE) yields fewer major complications at the cost of higher rebleeding rates, with overall mortality being broadly similar; this trade-off motivates strategies to improve durability in high-risk anatomy (2, 3).

The intermittent bleeding complicates decision making because catheter angiography may be negative at the moment of the study even when endoscopy or computed tomography angiography (CTA) has localized the culprit segment; in that therapeutic setting, empiric (“blind”), endoscopy-guided TAE achieves outcomes comparable to angiographically targeted embolization with procedure-related complications near 2%, confirming the safety of territorial occlusion when extravasation is not seen but the bleeding bed is known (4–8).

Distinct from empiric therapy during an ongoing bleed, prophylactic transarterial embolization (pTAE) refers to planned, early embolization after successful endoscopic hemostasis in high-risk patients, aimed at preventing rebleeding by diminishing arterial inflow beneath the ulcer bed (9).

This mini-review appraises pTAE after successful endoscopic hemostasis in NVUGIB, focusing on selection criteria, technical approach by arterial territory and embolic material, clinical outcomes, and complications.

## 2 Review literature summary

This review was conducted in accordance with the PRISMA guidelines. Since no human subjects or unpublished data were involved, Institutional Review Board approval was not required.

A comprehensive search was performed in PubMed and Scopus to identify studies published between January 2010 and September 2025. The strategy combined controlled vocabulary and free-text terms related to “upper gastrointestinal bleeding”, “gastrointestinal hemorrhage”, “transarterial embolization/embolization”, “angiographic treatment”, and “interventional radiology”, together with the terms “prophylactic”, “empiric/empirical”, “blind”, and “preventive”. Boolean operators (AND/OR) were applied to refine the search.

We considered original research articles (prospective or retrospective studies and randomized clinical trials) that reported technical and/or clinical outcomes of transarterial embolization for non-variceal upper gastrointestinal bleeding, specifically addressing the prophylactic approach, to be eligible for inclusion. We excluded case reports, conference abstracts, editorials, articles not written in English, studies primarily focused on variceal bleeding, and studies in which embolization was not performed or outcomes were not extractable. We also excluded studies that exclusively addressed empiric (‘blind’) embolization for angiography-negative bleeding, as well as reports in which the terms ‘prophylactic’ or ‘preventive’ were used to denote empiric embolization rather than planned post-endoscopic prophylaxis.

Systematic reviews and meta-analyses were not included in the selection, but they were considered in the discussion for context.

**Figure 1:**
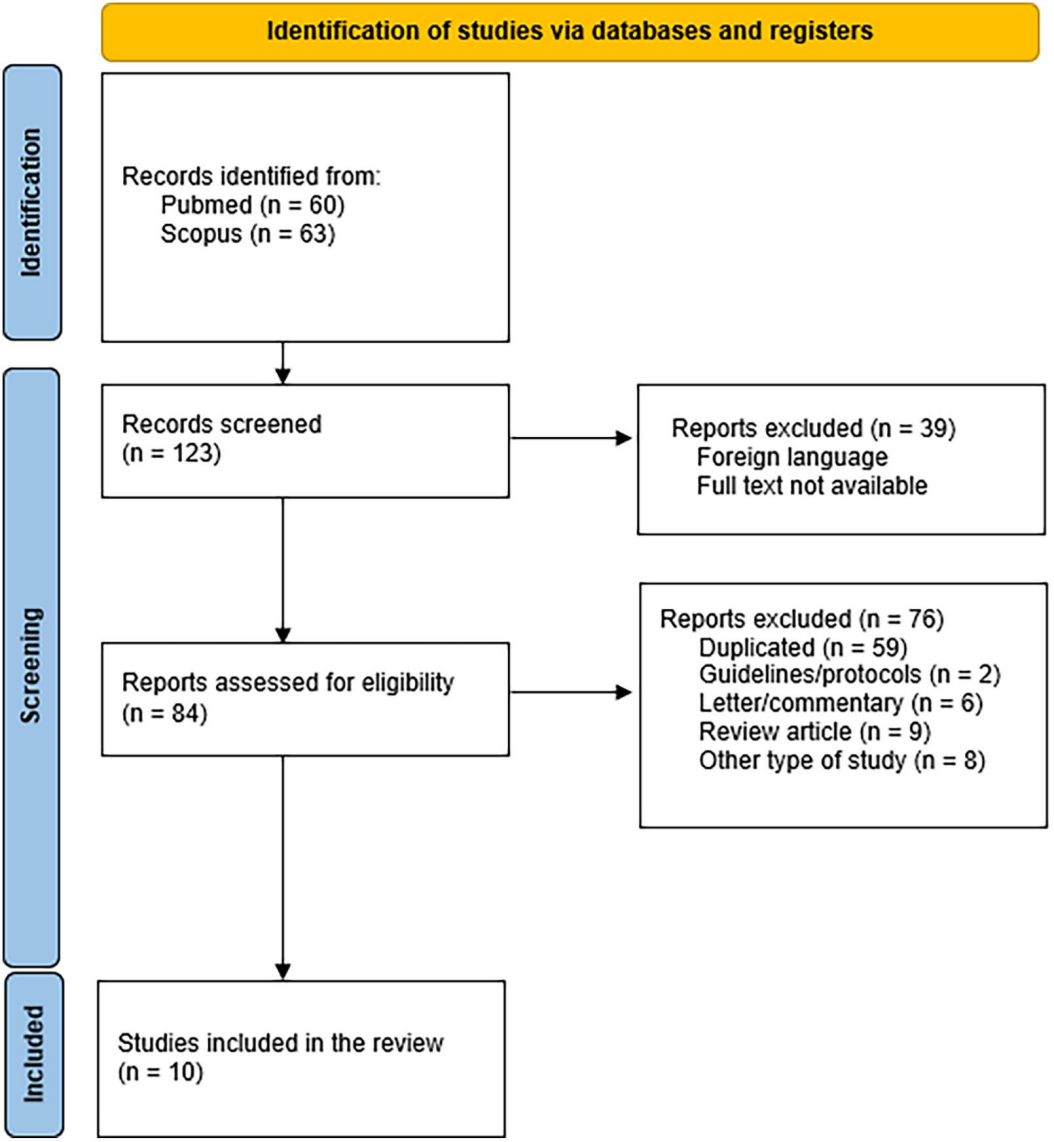
Flow-chart showing selection process of the articles included in the study.

The initial search retrieved 60 records from PubMed and 63 from Scopus. After screening titles and abstracts, 36 and 48 records were retained, respectively. Following the removal of duplicates, 59 unique studies remained. After full-text evaluation, 10 studies were finally included in this review: two randomized clinical trials (9, 10), three prospective studies (11–13), and five retrospective studies (14–18) (Figure 1).

The included table (Table 1) gives a summary of the recent literature presented throughout this review.

**Table 1:** Summary of the recent literature included in the review.

Authors, Year	Study type	N (total/groups)	Groups/Comparators	Indications/Selection	Target/Embolic	Technical success	Clinical success/Rebleeding	Complications	30-d Mortality
Alali et al., 2024 (14)	Retrospective series	10 (all prophylactic QS-GSP)	Single-arm prophylactic embolization (angiography-negative)	Suspected culprit territory after endoscopy/CTA; mixed sites incl. duodenum	GDA (3), jejunal (4), ileal (3); quick-soluble GSP ± coil	100%	90% clinical success; rebleeding 10% (salvaged by repeat TAE)	None procedure-related on follow-up	NR
Kaminskis et al., 2017 (11)	Prospective case–control	75 (25 P-TAE; 50 endoscopy alone)	Preventive angiography/TAE ≤24 h vs. endoscopy alone	Forrest I–IIb; Rockall ≥5	LGA or GDA by ulcer location; typical coil ± particles	NR	Rebleeding 12% (pTAE) vs. 22.4% (endoscopy)	No ischemic complications reported	4% vs. 16.3%
Kaminskis et al., 2019 (13)	Prospective cohort	399 high-risk (58 P-TAE; 341 endoscopy alone)	P-TAE within 24 h vs. endoscopy alone	Forrest Ia–IIb; Rockall ≥5 after endoscopic hemostasis	GDA/LGA per location	NR	3.4% (pTAE) vs. 16.2% (endoscopy) (*p* = 0.005)	No ischemic complications reported	5.7% vs. 8.5%
Kaminskis et al.,2017, Gastroenterol Res. (12)	Prospective case–control	111 total (37 P-TAE; 74 endoscopy alone); subset Rockal ≥7: 21 vs. 34	P-TAE ≤24 h vs. endoscopy alone	Forrest I–IIb; Rockall ≥5, subgroup ≥7	GDA/LGA per site	NR	Overall 11% (pTAE) vs. 27% (endoscopy) (*p* = 0.047); Rockall ≥7: 4.8% vs. 33%	No ischemic complications reported	5% vs. 11% (overall)
Lau et al., 2018 (9)	Multicenter RCT	241 (118 P-TAE; 123 control)	P-TAE after endoscopic hemostasis vs. standard care (both high-dose IV PPI)	Forrest Ia OR ulcer ≥20 mm OR Hb <9 g/dL OR shock; clip-guided; ≤ 12 h to angio	GDA or LGA; sandwich (distal coils + gelatin + proximal coils); empiric allowed if DSA negative	NR (96/118 underwent embolization)	30-d rebleeding ITT 10.2% vs. 11.4%; PP ≥15 mm: 4.5% vs. 23.1%	No ischemic complications reported	2.5% vs. 4.1% (ITT)
Laursen et al., 2014 (10)	Single-center RCT	105 (49 P-TAE; 56 control)	Supplementary embolization ≤24 h vs. standard; clip-guided; blind GDA allowed if clip lost	Forrest Ia–IIb peptic ulcers; post-endoscopic hemostasis	Culprit territory; coils	NR (18/49 randomized did not receive P-TAE)	Rebleeding ITT 2/49 vs. 8/56 (*p* = 0.10); adjusted *p* = 0.079	Partial coil displacement into hepatic branch; No ischemic complications reported	NR
Mille et al., 2015 (15)	Retrospective cohort	117 total (55 P-TAE; 47 conservative; 15 immediate TAE)	High-risk → P-TAE; others conservative; refractory → immediate TAE	Posterior bulb; Forrest I–IIc; Rockall ≥6 plus clinical/endoscopic risk	GDA; distal-to-proximal coils ± NBCA	98% (P-TAE)	Clinical success 87%; early rebleeding 11% (all salvaged)	Major 4% (1 pancreatitis; 1 fatal hepatic injury from coil migration); minor 15%	pTAE UGIB-related 4%
Nykänen et al., 2017 (16)	Retrospective case–control	85 (42 TAE; 43 surgery); P-TAE subset 16/42	TAE vs. surgery; P-TAE not analyzed separately	Bleeding gastric/duodenal ulcers after failed endoscopy	Selective; materials varied	NR	30-d rebleeding 25% (TAE) vs. 16.3% (surgery)	30-d complications 37.5% (TAE) vs. 67.4% (surgery)	12.5% (TAE) vs. 25.6% (surgery)
Ozen et al., 2025 (17)	Retrospective cohort	87 (pTAE vs. tTAE)	Prophylactic after endoscopic success vs. therapeutic after failure	Gastroduodenal peptic ulcer bleeding	Selective celiac/SMA; microcoils distal-to-proximal	NR	Overall 15%; pTAE 12% vs. tTAE 31% (ns)	23% any; major 7%; procedure-related deaths 3%	Overall 22%; pTAE 20% vs. tTAE 31% (ns)
Zetner et al., 2024 (18)	Retrospective cohort	176 (all P-TAE)	Standard vs. non-standard technique (internal comparison)	Post-endoscopic peptic ulcer bleeding; median Rockall 7	Standard: coiling GDA + side branches ± active site	NR	Rebleeding 25% overall; ↑ with non-standard (OR 3.30)	No ischemia/perforation	15% overall; ↑ with non-standard (OR 3.34)

P-TAE, prophylactic transarterial embolization; TAE, transarterial embolization; tTAE, therapeutic TAE; QS-GSP, quick-soluble gelatin sponge particles; GDA, gastroduodenal artery; LGA, left gastric artery; DSA, digital subtraction angiography; NBCA, N-butyl cyanoacrylate; RCT, randomized controlled trial; IV, intravenous; PPI, proton-pump inhibitor; OR, odds ratio; HR, hazard ratio; CI, confidence interval; NR, not reported; UGIB, upper gastrointestinal bleeding.

### 2.1 When to perform pTAE

Selection for pTAE is made based on ulcer size, clinical risk indices, and arterial topography.

Across the available trials and cohorts, pTAE was considered only after successful endoscopic haemostasis, typically achieved at the endoscopy using combination therapy (dilute epinephrine/adrenaline injection plus contact thermal coagulation and/or hemoclips).

A multicenter randomized controlled study enrolled 241 individuals with bleeding gastroduodenal ulcers who had achieved endoscopic hemostasis through hemoclipping or contact thermocoagulation with or without pre-injection of dilute epinephrine, and met at least one high-risk feature-spurting bleeding (Forrest Ia), ulcer diameter ≥20 mm, hemoglobin <9 g/dL, or shock-and allocated them to supplementary angiography and embolization within 12 h vs. standard care; both arms received high-dose intravenous proton-pump inhibition (9).

In the intention-to-treat analysis, 30-day rebleeding rates were 10.2% with assigned embolization and 11.4% with standard care (hazard ratio, 1.14; 95% CI, 0.53–2.46). Among the 96 cases who underwent embolization, rebleeding was 6.2% vs. 11.4% in controls, and ulcer size modified effect: for lesions ≥15 mm, per-protocol rebleeding fell to 4.5% after P-TAE compared with 23.1% under standard care, implying a number-needed-to-treat close to five; transfusion exposure decreased, and no embolization-related ischemia was reported (9).

A second randomized single-center study evaluated 105 patients with Forrest Ia–IIb peptic ulcers who underwent combined endoscopic therapy (1:10,000 diluted epinephrine injection, heat probe treatment, and/or hemoclip) followed by a 72-hour esomeprazole infusion. The study considered supplementary embolization within 24 h vs. standard care, using hemoclips to guide coil deployment and permitting “blind” GDA coiling when the clip had fallen off and no extravasation was seen; eighteen of 49 allocated to embolization did not undergo the procedure, mostly due to interventional radiology availability, yet intention-to-treat rebleeding occurred in 2/49 vs. 8/56 (*p* = 0.10), with multivariable adjustment favoring pTAE at trend level and a crude median length-of-stay reduction from 6 to 4 days (*p* = 0.028), while adverse events were uncommon and included a partially displaced coil into a hepatic branch without downstream injury (10).

Considered together, these trials define the operational features of pTAE - early, clip-guided, territory-based embolization after endoscopic control - and suggest that any aggregate benefit is contingent upon selection based on ulcer size and clinical status, rather than routine application (9, 10).

Case-control study that allocated Forrest I–IIb ulcers with Rockall ≥5 to preventive angiography/TAE within 24 h vs. endoscopy alone reported surgery in 8% vs. 35% (*p* = 0.012), rebleeding 12% vs. 22.4% (*p* = 0.358), and in-hospital mortality 4% vs. 16.3% (*p* = 0.258), with similar ICU and hospital stays and a higher ICU admission rate in the P-TAE arm (11).

Extending this approach across 2010–2017, the same authors reported that rebleeding was 11% with P-TAE (4/37) vs. 27% without (20/74; *p* = 0.047) and rescue surgery 2.7% vs. 23% (*p* = 0.017); when the subgroup with Rockall score ≥7 was analyzed, rebleeding was 4.8% with P-TAE compared with 33% after endoscopy alone, and no pTAE patient required surgery vs. 32% of controls (*p* = 0.004) (12).

A five-year series restricted to patients with Forrest Ia–IIb and Rockall ≥5, after primary endoscopic hemostasis, corroborated these findings by comparing a pTAE cohort with a contemporaneous control group consisting of similar high-risk patients who only underwent endoscopic hemostasis or who did not agree to undergo P-TAE, with comparable comorbid conditions and a similarly high predicted risk of rebleeding after endoscopic hemostasis; rebleeding rate was 3.4% (2/58) after prophylaxis vs. 16.2% (55/341; *p* = 0.005), with fewer units of fresh frozen plasma (1.3 ± 1.0 vs. 2.6 ± 1.7; *p* = 0.001), more packed red cells (6.6 ± 2.2 vs. 3.6 ± 2.5; *p* = 0.002), similar mortality (5.7% vs. 8.5%; *p* = 0.417), and no ischemic complications (13).

In their retrospective cohort, Mille et al. analyzed a structured pathway for managing bleeding duodenal ulcers, in which high-risk patients -defined as those with a posterior duodenal bulb ulcer with bleeding stigmata (Forrest I–IIc) and an estimated Rockall score ≥6, who additionally had ≥1 endoscopic risk factor (active bleeding or ulcer size ≥1 cm) and ≥1 clinical risk factor (hemodynamic instability, including systolic blood pressure <100 mmHg; age >80 years; ≥ 2 anticoagulants; or ≥2 comorbidities) - underwent prophylactic GDA embolization (performed within 24 h) at a mean 4 h 33 min after endoscopy, achieving technical success of 98% and 30-day clinical success of 87%; early rebleeding occurred in 11% with complete salvage by repeat therapy, no late rebleeding was observed, major complications occurred in 4% (including one fatal hepatic injury due to coil migration in a cirrhotic patient), and minor device-related events in 15%; across the entire 117-patient cohort, surgery for ulcer bleeding was required in 0.9% (15).

Recent single-center studies emphasize that workflow and technique have a strong influence on outcomes.

In 176 patients treated with prophylactic GDA embolization after endoscopic control, rebleeding and 30-day mortality were 25% and 15%, respectively; non-standard technique - deviation from a predefined template coiling the GDA and its side branches, together with treatment of any active bleeding focus - tripled the odds of rebleeding (OR 3.30, 95% CI 1.48–7.33) and of 30-day mortality (OR 3.34, 95% CI 1.27–8.77), whereas more than one endoscopy before TAE independently predicted rebleeding; ischemia or perforation did not occur (18).

A retrospective cohort that explicitly distinguished prophylactic TAE after endoscopic success from therapeutic TAE for failed hemostasis reported overall rebleeding 15% and 30-day mortality 22%, with non-significantly lower rebleeding in prophylaxis (12% vs. 31%) and mortality (20% vs. 31%). In that protocol, Forrest I–IIb ulcers underwent combination endoscopic therapy (13–25 mL adrenaline–saline injection followed by thermal probe/APC or hemoclips) with Rockall scoring; TAE was performed without delay if primary hemostasis failed, whereas prophylactic TAE was considered case-by-case after successful hemostasis in high-risk patients receiving high-dose IV PPI. ASA IV independently predicted death (HR 3.43, 95% CI 1.52–7.71), duodenal location correlated with worse survival (HR 6.01, 95% CI 1.24–29.28), and procedure-related complications occurred in 23%, including major 7% and procedure-related deaths 3% (17).

### 2.2 How to perform pTAE: target territory and embolic materials

The technical approach should reflect arterial supply and aim to reduce territorial influx.

Target selection follows ulcer site: GDA for duodenal, antral, or pyloric ulcers and LGA for lesser-curvature, fundal, or angulus ulcers (9, 13).

A distal-to-proximal approach -deploying distal coils beyond the ulcer bed, then coiling the proximal trunk, followed by superior mesenteric and inferior pancreaticoduodenal angiography to detect and treat retrograde arcade inflow, reduces the likelihood of back-door reperfusion, a key consideration in the gastroduodenal arcade (9, 18–20).

The choice of embolic material should reflect the flow dynamics and coagulation status. Coils alone are discouraged in high-flow vessels or when coagulation is impaired; coil–gelatin sponge combinations are suitable for many preventive cases. Liquid agents, such as N-butyl-cyanoacrylate or EVOH/Onyx, provide rapid, coagulation-independent occlusion when operator expertise is present (21, 22).

In the prophylactic setting, absence of angiographic extravasation is expected, and territory-guided embolization is appropriate; this was recommended within the trial protocol using a distal-to-proximal “sandwich” even when angiography was negative (9).

Ozen et al. reported that rebleeding was significantly associated with coil burden, with most rebleeding events occurring in the 13–25 coils category (*p* = 0.040), suggesting that cases requiring more extensive coil packing may represent a higher-risk subgroup (17).

For angiography-negative but well-localized scenarios in which theoretical ischemia is a concern, quick-soluble gelatin sponge particles that resorb within four hours achieved 100% technical success and 90% short-term clinical success with 10% rebleeding and no embolization-related complications in a small series that included duodenal ulcers, suggesting a niche strategy that merits further evaluation (14).

### 2.3 Outcomes and complications

Regarding clinical outcomes, Lau et al, in their randomized evaluation, did not show an overall reduction in 30-day rebleeding on an intention-to-treat analysis; however, a plausible size-based effect was identified in ulcers ≥15 mm, with reduced transfusion exposure and no embolization-related ischemia among treated patients (9).

Kaminskis et al. demonstrate lower rebleeding and fewer operations with pTAE (11% vs. 27% and 2.7% vs. 23% across matched comparators) and, in Rockall ≥7, rebleeding 4.8% with pTAE vs. 33% without and 0% vs. 32% requiring surgery, without excess mortality or ischemic events, indicating that properly targeted inflow reduction can translate into clinically relevant durability (11–13).

In a series treating duodenal ulcer bleeding, early rebleeding occurred in 11% of cases with no late recurrences. Major complications were seen in 4%-including a liver injury from coil migration-highlighting the risk near the common hepatic origin and the need for meticulous technique (15).

Zetner et al. reported, in a recent single-center experience, that ischemia or perforation did not occur, despite a rebleeding rate of 25% and a mortality rate of 15%.

Outcomes were more strongly associated with adherence to a standardized treatment protocol than with lesion-level variables or visualization of active bleeding on angiography (18).

Once prophylactic and therapeutic embolization were analyzed separately, the prophylactic group showed numerically lower rebleeding and 30-day mortality, with similar complication rates. Overall mortality, however, was determined by physiological reserve, as illustrated by the impact of ASA IV status (17).

Endovascular therapy compared with surgery shows similar mortality rates, higher rebleeding rates, and substantially fewer major complications. This observation supports the safety envelope for interventional strategies and suggests that preventing recurrence in the highest-risk anatomy could improve overall outcomes without incurring surgical risks (2).

Finally, comparative surgical data after failed endoscopy showed similar 30-day mortality between TAE and surgery, fewer complications with TAE, and the presence, but not a separate analysis, of a pTAE subset within the endovascular arm, situating pTAE within a pathway that privileges endovascular safety while acknowledging rebleeding as the principal trade-off (16).

## 3 Discussion

The evidence reviewed suggests that p-TAE after effective endoscopic hemostasis is technically feasible in most centers, generally safe in the gastroduodenal and left gastric territories, and potentially helpful when careful patient selection and timing of embolization are employed. Across randomized and observational studies, any apparent benefit is concentrated in patients enriched for large ulcers, high clinical risk scores, and unfavorable arterial topography, whereas procedural delay and non-standardized embolization techniques appear to attenuate efficacy.

A meta-analysis by Chang et al. provides an important synthesis of the field (23). That review included five eligible studies, of which four were comparative (2 randomized and 2 retrospective cohort studies) and one was a single-arm cohort. Overall, 310 patients underwent prophylactic TAE, and 255 of these were compared with 580 controls treated with endoscopic therapy alone. In pooled comparative analyses, prophylactic TAE was associated with lower 30-day or in-hospital rebleeding (OR 0.35, 95% CI 0.15–0.85) and lower mortality (OR 0.28, 95% CI 0.10–0.83), whereas the reduction in reintervention did not reach statistical significance (OR 0.68, 95% CI 0.43–1.08) (23). The pooled technical success rate was 90.5%, and the overall complication rate was very low, with no ischemic complications reported in the included studies. However, these favorable findings should be interpreted with caution because the evidence base was limited, the pooled sample was relatively small, and the analysis relied substantially on non-randomized data (23).

A mixed-design meta-analysis pooling three randomized and nine observational studies (*n* = 1,329; 486 received P-TAE), comparing prophylactic embolization after endoscopic hemostasis vs. standard care without prophylactic embolization, reported lower odds of rebleeding with prophylaxis (OR 0.48, 95% CI 0.29–0.78), fewer any reinterventions (OR 0.48, 95% CI 0.31–0.76) and fewer surgeries (OR 0.35, 95% CI 0.14–0.92), without a difference in mortality; in randomized-only intention-to-treat analyses the effect did not reach statistical significance, whereas per-protocol analyses favored P-TAE, and sequential evidence monitoring suggested that the randomized evidence remains underpowered and not yet definitive (24).

An updated review, including ten studies (*n* = 1,253), did not demonstrate a statistically significant overall reduction in rebleeding vs. standard care (OR 0.69, 95% CI 0.39–1.22; I^2^ = 27%) or in mortality (OR 0.70, 95% CI 0.40–1.23), and found no differences between P-TAE and therapeutic TAE for rebleeding (OR 1.08, 95% CI 0.70–1.68); adverse events were uncommon and comparable, while any suggestion of benefit emerged mainly in subgroups with very large ulcers, whereas the overall pooled effect remained non-significant (25).

The most conservative assessment comes from the recent Cochrane review, which combined the available randomized trials only.

In that analysis,pTAE did not show a statistically significant reduction in 30-day rebleeding(OR 0.58, 95% CI 0.18–1.83), reintervention per event (OR 0.68, 95% CI 0.35–1.35) or per patient (OR 0.65, 95% CI 0.25–1.69), or early mortality (OR 0.41, 95% CI 0.14–1.21), while hospital stay may be shorter by a mean 2.41 days (95% CI −4.06 to −0.76). Sensitivity analyses restricted to high-risk stigmata (Forrest Ia–IIa) did not materially change these conclusions, and the overall certainty of evidence was rated low because of small sample size, lack of blinding, protocol violations, potential selection bias, and incomplete reporting of non-bleeding adverse events. Taken together, these data indicate that the current randomized evidence remains underpowered and insufficient to support routine prophylactic embolization for all high-risk presentations (26).

Current guideline positions are broadly aligned with this interpretation. The American Gastroenterological Association does not endorse routine prophylactic embolization after endoscopic hemostasis, recommends repeat endoscopy first for recurrent bleeding, and reserves TAE or surgery for refractory cases individualized to etiology, hemodynamics, comorbidity, and local expertise; when angiography is negative, clip-guided territorial embolization is framed as therapeutic empiric care rather than prophylaxis (27).

From a practical standpoint, management should remain anchored to the endoscopic outcome., Failure of endoscopic hemostasis leads to therapeutic embolization (tTAE), whereas pTAE should be considered only after successful hemostasis in carefully selected high-risk patients. This distinction is clinically important and should also be maintained conceptually in the literature.

In this regard, greater terminological precision is essential.

Empiric TAE-undertaken during the index hemorrhage when catheter angiography is negative but endoscopy or computed tomography angiography localizes the culprit bed, demonstrates hemostatic outcomes comparable to targeted TAE with 2% embolization-related complication rates and provides a rational option for angiography-negative therapeutic management; however, these data are not a surrogate for prophylactic benefit after endoscopic hemostasis and should be appraised separately (4).

A pragmatic interpretation of the available literature is therefore that pTAE may be reasonable when several high-risk features converge: ulcer size ≥15–20 mm, Rockall enrichment (≥5 and particularly ≥7), and ulcer location over major arterial territories such as the gastroduodenal artery or left gastric artery. In such settings, benefit is more likely when embolization is performed early (ideally within 12–24 h) and according to a standardized territorial approach, including distal-to-proximal occlusion, assessment of collateral inflow, and embolic selection tailored to flow dynamics and coagulation status. Conversely, the absence of a consistent overall benefit in randomized analyses argues strongly against indiscriminate routine prophylaxis (9, 11, 13, 15, 18, 21).

Overall, the literature supports a selective, anatomy- and risk-based role for pTAE rather than universal adoption. The key unresolved issue is not whether pTAE can work, but which patients benefit sufficiently to justify an additional invasive procedure after successful endoscopic hemostasis. Future multicenter randomized trials should therefore use explicit size- and score-based entry criteria, standardized embolization templates, strict timing targets, and comprehensive adverse-event reporting. Only with this level of methodological consistency will it be possible to define the true clinical value of preventive embolization and identify the subgroup in whom pTAE provides meaningful incremental benefit (26, 27).

## 4 Conclusions

In conclusion, pTAE is feasible and generally safe in experienced centers, and it appears to reduce rebleeding and the need for surgical rescue in well-selected populations. However, randomized data do not support routine application to all high-risk presentations and are not endorsed by guidelines.

Therefore, the development of new randomized trials is necessary, with explicit inclusion based on size and scores, uniform technique, timely intervention, standardized endpoints, and comprehensive reporting of adverse events to precisely define who benefits most and how best to administer prophylaxis on a large scale.
